# Combinational zimberelimab plus lenvatinib and chemotherapy for alpha-fetoprotein elevated, advanced gastric cancer patients (AFPGC): a phase 1 dose-escalation study

**DOI:** 10.1007/s00262-024-03743-0

**Published:** 2024-06-04

**Authors:** Ting Deng, Feixue Wang, Le Zhang, Tao Ning, Yansha Sun, Shaohua Ge, Ming Bai, Yao Lu, Hongli Li, Yi Ba

**Affiliations:** 1 Department of GI Medical Oncology, Tianjin Medical University Cancer Institute and Hospital, National Clinical Research Center for Cancer, Tianjin’s Clinical Research Center for Cancer, Tianjin Key Laboratory of Digestive Cancer, Key Laboratory of Cancer Prevention and Therapy, Tianjin Medical University, Tianjin, China; 2 Department of Medical Oncology, Department of Cancer Center, Peking Union Medical College Hospital, Beijing, China

**Keywords:** Gastric cancer, AFP, Immunotherapy, Phase 1 trial

## Abstract

**Background:**

Alpha-fetoprotein elevated gastric cancer (AFPGC) got growing interests for its aggressive nature and unfavorable prognosis. Here, a phase 1 dose escalation study was conducted to evaluate safety and efficacy of zimberelimab (GLS-010, anti-PD-1) plus lenvatinib and chemotherapy (XELOX) as the first-line treatment for AFPGC.

**Methods:**

Histologically confirmed HER2-negative, advanced GC patients with elevated serum AFP level (≥ 20 ng/ml) were screened. Using a 3 + 3 dose escalation design, patients were administered varying doses of lenvatinib (12, 16, 20 mg) with GLS-010 and XELOX. The primary endpoints were safety and determination of recommended phase II dose (RP2D). Secondary endpoints included overall response rate (ORR), progression-free survival (PFS) and disease control rate.

**Results:**

Nine patients were enrolled with no dose-limiting toxicities observed. Most frequent treatment-related AEs were fatigue (55.6%), hand-foot syndrome (55.6%) and rash (55.6%), and no grade ≥ 4 AEs were reported. All patients exhibited disease control with ORR reaching 33.3%. The median PFS and OS reached 7.67 months (95% CI 4.07–11.27) and 13.17 months (95% CI 2.78–23.56), respectively. Serum AFP level was found correlated with therapeutic responses. Further 16s rRNA sequencing analysis demonstrated altered gut microbiota with elevated abundance of *Lachnospiraceae bacterium-GAM79* and *Roseburia hominis A2-183.*

**Conclusions:**

GLS-010 plus lenvatinib and XELOX demonstrated a manageable safety profile with promising efficacy for AFPGC. With RP2D of lenvatinib determined as 16 mg, further expansion cohort is now ongoing. Translational investigation suggested that serum AFP can be indictive for therapeutic responses and certain microbiota species indicating favorable responses to immunotherapy was elevated after the combinational treatment.

**Supplementary Information:**

The online version contains supplementary material available at 10.1007/s00262-024-03743-0.

## Introduction

Last decades have witnessed a growing interest in alpha-fetoprotein (AFP)—producing gastric cancer (AFPGC), also known as AFP-positive or AFP-elevated gastric cancer, due to its aggressive phenotype and poor prognosis. With no consensus reached on accurate definition and cut-off value of AFP level, a proportion ranging from 2.9 to 19.1% has been reported by different literature [1–4]. Currently, our understanding toward AFPGC mainly comes from retrospective analyses, data on therapeutic exploration are even less. Following current standard for general population, clinical outcomes are far from satisfactory.

Much effort has been devoted to improve the therapeutic efficacy and survival of AFPGC. Based on the observations that VEGF expression is higher in AFPGC compared with general GC population [5], novel targeted regimen was speculated to be possibly effective. Apatinib, an oral tyrosine kinase inhibitor, was evaluated in previously treated AFPGC and got promising survival data [6]. Moreover, stronger combination pattern of apatinib with Camrelizumab (anti-PD-1) and SOX reported an objective response rate (ORR) of 55.6% [7], highlighting the great promise of adding targeted and immune reagents to traditional chemotherapy in AFPGC. Lenvatinib, a multi-kinase inhibitor of angiogenic (including VEGF receptor) and oncogenic receptor tyrosine kinases, can exert immunomodulatory effects via inhibiting the infiltration of immunosuppressive cells (Treg cells) and activating cytotoxic CD8^+^ T cells [8]. Thus, combining multiple receptor tyrosine kinase inhibitor (TKI) with immune checkpoint inhibitors (ICIs) is expected to enhance anti-cancer immunity, which has already been proved by preclinical and clinical studies [9–11]. Combining lenvatinib with pembrolizumab (anti-PD-1) has already demonstrated clinical efficacy in various solid tumors [12, 13], including gastric cancer [14]. Given molecular foundation and clinical evidence mentioned above, combinational Lenvatinib with anti-PD-1 and chemotherapy provides an attractive strategy for AFPGC treatment.

In this study, a single-center, prospective dose-escalation study was conducted to evaluate the safety and clinical efficacy of combining zimberelimab (GLS-010, anti-PD-1) with lenvatinib and chemotherapy (XELOX) as first-line treatment for patients with AFP-elevated, HER2-negative, advanced gastric adenocarcinoma. A 3 + 3 dose escalation design was employed to determine the recommended phase II dose (PR2D) for lenvatinib in this combination pattern (ClinicalTrials.gov Identifier: NCT05221775).

## Materials and methods

### Study design and participants

An open-label, single-center, phase I trial was conducted at Tianjin Medical University Cancer Institute and Hospital to evaluate the safety and efficacy of zimberelimab (anti-PD-1 antibody, GLS-010) plus lenvatinib with chemotherapy (XELOX) in AFP-elevated and HER2-negative advanced gastric and gastroesophageal junction (G/GEJ) adenocarcinoma in the first-line setting. A traditional 3 + 3 escalation design was employed to determine the safety, tolerability, maximum tolerated dose (MTD), and recommended RP2D of lenvatinib when combined with GLS-010 and XELOX, starting at a dose of 12 mg.

Patients included in the trial met the following criteria: over 20 years old, histologically confirmed unresectable or metastatic G/GEJ adenocarcinoma, elevated serum AFP levels (≥ 20 ng/ml) at diagnosis, centrally confirmed HER2-negative, with measurable disease according to Response Evaluation Criteria in Solid Tumors (RECIST) criteria (version 1.1), no prior anticancer treatment, and adequate organ function (hemoglobin ≥ 9.0 g/dL; absolute neutrophil count ≥ 1,500 cells/mL; platelet count ≥ 90,000 cells/mL; total bilirubin concentration ≤ 1.5 × upper limit of normal [ULN] or direct bilirubin ≤ ULN; AST and ALT concentrations ≤ 2.5 × ULN or ≤ 5 × ULN for patients with liver metastasis; and calculated creatinine clearance > 60 mL/min or creatinine ≤ 1.5 × ULN). Patients were also required to have an Eastern Cooperative Oncology Group (ECOG) performance status score of 0–2. The exclusion criteria were as follows: recent systematic anti-cancer treatments (chemotherapy, radiotherapy, immunotherapy or targeted therapy for G/GEJ cancer) within 2 weeks, grade 3–4 gastrointestinal bleeding within 3 months or arterial thromboembolic events (including but not limited to myocardial infarction, transient ischemic attack, or unstable angina) within 6 months, uncontrolled hypertension, active central nervous system (CNS) metastasis or carcinomatous meningitis, HIV infection or hepatitis B or C infection history, autoimmune diseases, cardiovascular disease (including myocardial infarction, congestive heart failure, severe or unstable angina) and secondary malignancy with certain exceptions (adequately treated in situ carcinoma of any organ, basal cell carcinoma of the skin). The study received approval from the ethics committee and institutional review boards of Tianjin Medical University Cancer Institute and Hospital. Written informed consent was obtained from all participants.

### Treatments

Following necessary premedication, enrolled patients were treated with zimberelimab (GLS-010), lenvatinib and chemotherapy (XELOX). This 21-day therapeutic cycle entailed an intravenous infusion of GLS-010 at a dosage of 240 mg on day 1, along with XELOX (oxaliplatin 130 mg/m^2^, day 1; capecitabine 850–1250 mg/m^2^, twice daily, day 1–14), and lenvatinib (12 mg/16 mg/20 mg, once daily, day 1–21). The starting dosage of 12 mg for lenvatinib was determined based on previous publications and drug instruction [14, 15]. Treatment was scheduled for 6 cycles, after which patients would enter the maintenance stage with GLS-010 plus lenvatinib and capecitabine until disease progression, development of toxic events, initiation of another anticancer treatment, or other specified discontinuation criteria outlined in the protocol.

### Outcomes

The primary endpoint was to evaluate the safety of the combination treatment and determine RP2D of lenvatinib. Adverse events (AEs), serious AEs (SAEs), and dose limiting toxicities (DLT) of lenvatinib were calculated according to National Cancer Institute Common Terminology Criteria for Adverse Events (NCI-CTCAE) version 4.02. The DLT observation period encompassed the first two treatment cycles. The secondary endpoints included ORR, disease control rate (DCR), PFS, overall survival (OS) and duration of response (DoR). Radiographic screening was conducted every 2 treatment cycles (6–8 weeks). The therapeutic efficacy was categorized per RECIST version 1.1 criteria into complete response (CR), partial response (PR), stable disease (SD), or progressive disease (PD). For patients evaluated as CR or PR, a secondary radiographic assessment was required in the subsequent cycle for validation.

### 16s rRNA sequencing analysis

Stool samples were collected at baseline (enrollment) and after the investigated treatments to gain deeper insights into gut microenvironment alternation during treatments. According to manufacturer’s protocols, microbial DNA was extracted from fecal samples using E.Z.N.A.® soil DNA Kit (Omega Bio-tek, Norcross, GA). Following standard PCR amplification, sequencing analysis was conducted on the Illumina MiSeq PE300 platform (Illumina, San Diego, USA). General statistical analysis was performed using R (version 4.1.3). Principal component analysis (PCA), based on Bray–Curtis matrices, was conducted to assess the differences in beta diversity among groups, and statistical significance was determined by permutational multivariate analysis of variance (PERMANOVA). To compare the relative abundance of different taxa between groups, nonparametric Kruskal–Wallis test was used. Then, linear discriminant analysis (LDA) effect size (LEfSe) method was conducted with a *p* value < 0.05 for the Kruskal–Wallis test and a size-effect threshold at 2.0 on the logarithmic LDA score.

### Single cell RNA (scRNA) sequencing

Gastric cancer tissue was obtained by endoscopic biopsy, with written informed consent obtained before sampling. Following a standard protocol, single cell suspension was prepared and then loaded onto a Singleron GEXSCOPE instrument. According to manufacturer’s instructions, 8000 single cells were captured. The subsequent steps of cDNA amplification and library construction were performed according to the standard protocol of Singleron Biotechnologies. Inc. (Nanjing, China), ensuing a minimum depth of 20,000 reads per cell. With principal component analysis (PCA) and Louvain algorithm, the cells were separated into multiple clusters. Seurat software was employed to identify upregulated genes in different cell clusters, and based on unbiased cell type recognition, certain cell types were finally determined. CeleScope software (https://github.com/singleron-RD/CeleScope) was used to analyze the sequencing data. CellPhoneDB (v.1.1.0) was adopted to predict the pairwise interactions between cell clusters.

### Statistical analysis

The sample size for this study was determined based on the standard 3 + 3 dose-escalation design. Patients who received at least one dose of regimen were included in final analysis. Descriptive statistics were used for patient clinicopathological characteristics, safety, and response data. Median PFS with a 95% confidence interval (CI) was calculated using Kaplan–Meier method. The calculations of geometric mean, normal mean, median, and interquartile range were performed using SPSS® version 25.0 (IBM, Armonk, NY, USA).

### Data availability

All clinical trial data presented in the article are not publicly available, as it could compromise patient privacy or consent, but are available upon reasonable request by contacting the corresponding author. Use of data must comply with the requirements of Human Genetics Resources Administration of China and other country or region-specific regulations.

## Results

### Patients and treatment

Between November 2021 and May 2024, a total of 9 patients were enrolled under 3 + 3 dose escalation design. No DLTs were observed across the three dosage groups. Enrolled patients had a median age of 58 years (range 35–73). As detailed in Table 1, apart from one patient who experienced disease progression after previous adjuvant chemotherapy, the remaining eight patients presented with advanced disease at initial diagnosis. Five patients exhibited two metastasis sites at enrollment, while three had over three dismal metastatic organs. The most common metastasis site observed in all patients was lymph nodes, followed by liver metastasis in five patients and lung metastasis in four. Moreover, bone metastasis and peritoneal metastasis were each identified in two patients at diagnosis. These findings support the malignant nature of AFPGC. The median serum AFP level was 743 ng/ml (mean, 19,826 ng/ml; SD, 56,781), with three patients exceeding 1000 ng/ml and three less than 100 ng/ml. Among the three patients over 1000 ng/ml, one participant exhibited an extremely high level of 171,211 ng/ml. After excluding the maximum, the mean AFP level was 903.3 ng/ml (SD, 1250).

**Table 1:** Baseline patient and disease characteristics

Characteristics	Number	%
*Age (years)*		
Median (range)	58(35–73)	
*Gender*		
Male	7	77.8
Female	2	22.2
*ECOG status*		
0	5	55.6
1	4	44.4
*Pathologic differentiation*		
Signet-ring cell carcinoma	1	11.1
Poorly differentiated adenocarcinoma	5	55.6
Moderately/poorly differentiated adenocarcinoma	3	33.3
*Location of primary tumor*		
Cardia or fundus	2	22.2
Body	2	22.2
Antrum or pylorus	1	11.1
Overlapping	4	44.4
*Serum AFP level at diagnosis*		
$\leq$100 ng/ml	3	33.3
100–1000 ng/ml	3	33.3
≥ 1000 ng/ml	3	33.3
*Previous adjuvant chemotherapy*		
Yes	1	11.1
No	8	88.9
*Number of metastatic organs at enrollment*		
1	1	11.1
2	5	55.6
≥ 3	3	33.3
*Metastatic sites at enrollment*		
Liver	5	55.6
Lung	4	44.4
Lymph nodes	9	100
Peritoneum	2	22.2
Bone	2	22.2

*AFP* alpha-fetoprotein, *ECOG* Eastern cooperative oncology group

As of the cutoff date of May 2024, one patient remained under treatment. Seven patients experienced disease progression and died mainly for cancer cachexia. One patient withdrew from the study for personal reasons after completing three treatment cycles. In total, the median duration for treatment was 6 cycles (range 3–6 cycles) with 2 maintenance cycles (range 0–17 cycles).

### Safety analysis

All participants experienced at least one drug-related AEs, with most AEs categorized as grade 1 or 2. No grade 4 or 5 AEs were reported. As shown in Table 2, the most frequently observed AEs were fatigue (55.6%), hand-foot syndrome (55.6%) and rash (55.6%), followed by neutropenia (44.4%), thrombocytopenia (33.3%) and leukopenia (33.3%). Two patients from 20 mg dosage group experienced grade 3 AEs. One participant suffered grade 3 hand-foot syndrome, while another experienced grade 3 hepatic dysfunction and myelosuppression (thrombocytopenia, leukopenia, and neutropenia). After symptomatic treatment, both patients recovered and continued their treatment with reduced dosage as per the study protocol. Furthermore, one patient developed grade 1 pneumonia identified by routine radiographic screening. With GLS-010 held for one cycle and prednisone (1 mg/kg, QD) administrated for five days, followed by half dose for another 5 days, obviously alleviated inflammatory infiltrates in chest CT screening was identified. No AE associated dropouts and deaths occurred in three dose groups.

**Table 2:** Any grade and grade ≥ 3 AEs in safety analysis

AE	12 mg (*N* = 3)		16 mg (*N* = 3)		20 mg (*N* = 3)		Total (*N* = 9)	
	All Grade	Grade ≥ 3	All Grade	Grade ≥ 3	All Grade	Grade ≥ 3	All Grade	Grade ≥ 3
*General disorders*								
Fatigue	1	0	2	0	2	0	5 (55.6%)	0
Gastrointestinal Reactions	0	0	1	0	1	0	2 (22.2%)	0
Hand-foot syndrome	2	0	1	0	2	1	5 (55.6%)	1(11.1%)
Hepatic injury	0	0	0	0	1	1	1 (11.1%)	1(11.1%)
Rash	2	0	0	0	3	0	5 (55.6%)	0
Hypertension	1	0	0	0	1	0	2 (22.2%)	0
*Myelosuppression*								
Anemia	0	0	0	0	1	0	1 (11.1%)	0
Neutropenia	1	0	2	0	1	1	4 (44.4%)	1(11.1%)
Leukopenia	1	0	1	0	1	1	3 (33.3%)	1(11.1%)
Thrombocytopenia	0	0	2	0	1	1	4 (44.4%)	1(11.1%)
*Immune-associated disorders*								
Pneumonia	1	0	0	0	0	0	1 (11.1%)	0
Hypothyroidism	1	0	1	0	0	0	2 (22.2%)	0

*AEs* adverse events

### Efficacy analysis

**Fig. 1:**
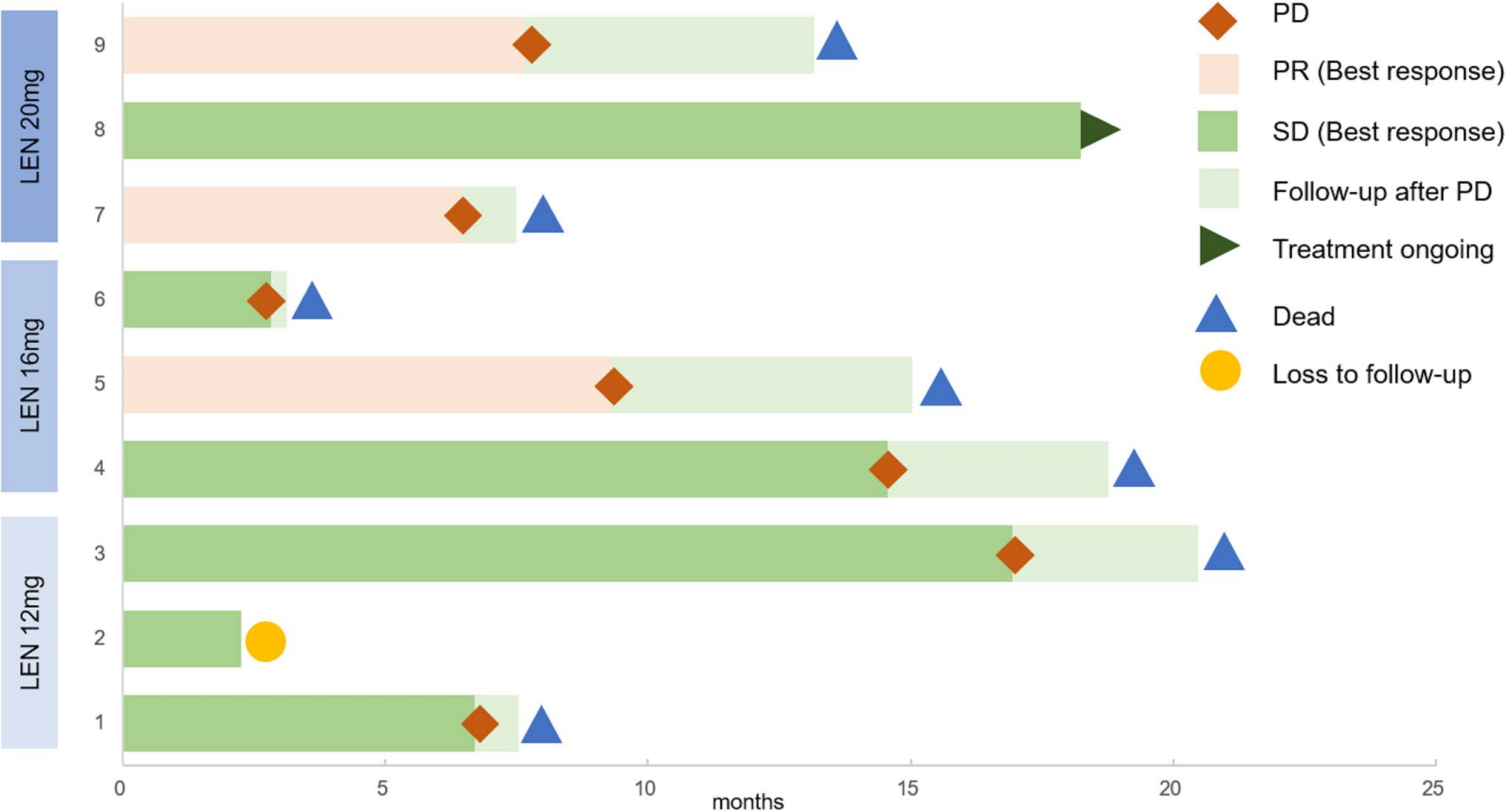
Follow-up and survival. Swimming plot illustrating events during treatment and follow-up (n = 9). *LEN* lenvatinib, *PD* progressive disease, *PR* partial responses, *SD* stable disease

One patient was still under treatment at the cutoff date, receiving GLS-010 plus lenvatinib and capecitabine as maintenance treatment (Fig. 1). Seven patients experienced disease progression and died, mainly for cancer cachexia. Additionally, one patient withdrew from study for personal reason after completing three cycles of therapy. Among all the enrolled, three cases of PR were observed, including one participant from 16 mg dosage group and two in 20 mg dosage (Table 3). The other six patients exhibited SD, with four experiencing tumor shrinkage of less than 30%. All patients exhibited disease control (DCR: 100%) and ORR reached 33.3%. Collectively, the mPFS and mOS reached 7.67 months (95% CI 4.07–11.27 months) and 13.17 months (95% CI 2.78–23.56 months), respectively.

**Table 3:** Treatment details and therapeutic efficiency analysis

Parameters	12 mg (*N* = 3)	16 mg (*N* = 3)	20 mg (*N* = 3)	Total (*N* = 9)
*Treatment information*				
Therapeutic cycles (median)	6	4	6	6
Maintenance cycles (median)	2	7	2	2
*Overall responses (%)*				
CR	0	0	0	0
PR	0	1	2	3
SD	3	2	1	6
PD	0	0	0	0
ORR	0	33.3	66.7	33.3
DCR	100	100	100	100
Median PFS (95% CI, months)	7.67 (4.07–11.27)			
Median DoR (95% CI, months)	13.17 (2.78–23.56)			

*CR* complete response, *DCR* disease control rate, *ORR* overall response rate, *PD* progressive disease, *PFS* progression-free survival, *PR* partial responses, *SD* stable disease

### Exploratory analysis

**Fig. 2:**
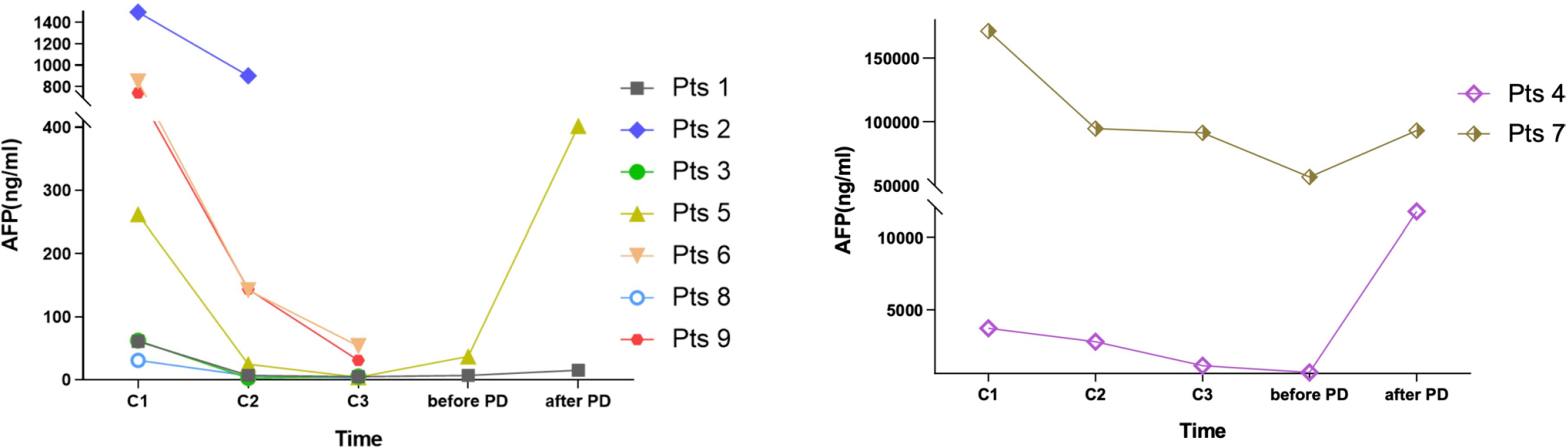
Dynamic monitoring of serum AFP level during treatment. *Pts* patients

To explore the predictive value of serum AFP level in clinical responses and prognosis, dynamic monitoring of serum AFP levels during treatment was performed (Fig. 2). After two cycles’ treatments, all participants experienced varying degrees of decline in AFP levels. Moreover, a rebound in AFP levels was observed around the timepoint of disease progression. While limited to sample size, no further statistical correlation was reached between baseline AFP levels and clinicopathological features, including tumor burden, clinical responses, and prognosis.

**Fig. 3:**
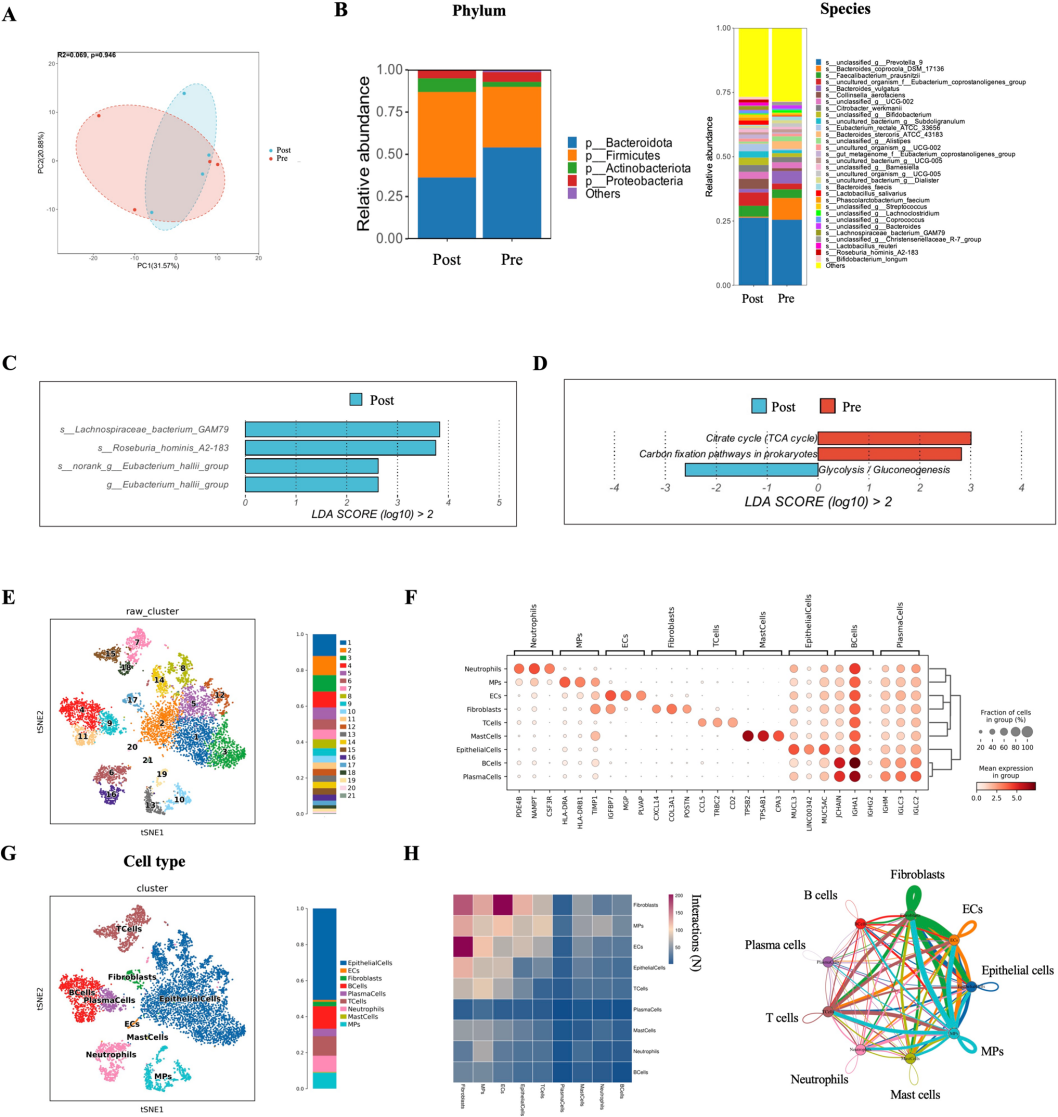
Fecal microbiota analysis and single-cell RNA sequencing analysis. **A** PCA based on bray–curtis matrices, revealing microbiota community composition at baseline (Pre, Red) and after treatment (Post, Blue). (*n* = 4 for Pre and Post group). **B** The relative abundance of fecal microbiota at phylum level and species level in patient fecal microbiota community. **C** Significantly enriched genus at baseline or after treatment analyzed by linear discriminant analysis effect (LDA effect, adjusted *p* $<$ 0.05). **D** Metabolic pathways significantly enriched by LDA effect at baseline or after treatment. **E** The t-distributed stochastic neighbor embedding (t-SNE) plot of the 21 main cell types identified from gastric cancer tissue. **F** Dot-plot illustrating the expression levels of cell type marker genes in 8 cell types. **G** t-SNE plot of cells from cancer tissue (colored by cell type). **H** Heatmap and circo plot representing the cell interactions predicted by CellphoneDB. The nodes with different colors represent cell types. The width of the edge represents the number of significant ligand-receptor pairs between the two cell types. Color of the edge is consistent with ligand cell types.

Stool samples were collected before and after the investigated treatments, and 16s rRNA sequencing was performed to provide an initial overview of gut microbiota alternation during treatments. PCA revealed the altered gut microbiota after combinational therapy though with no statistical significance (Fig. 3A). A phylum-level analysis observed increased abundance of *Firmicutes* and *Actinbacteria* (Fig. 3B)*.* At the species level, *Lachnospiraceae bacterium-GAM79, Roseburia hominis A2-183* and *Eubacterium hallii* exhibited significant elevation after treatment, as demonstrated by abundance analysis (Fig. 3B) and LEfSe (LDA Effect Size) analysis (Fig. 3C). Further gene function analysis through LEfSe uncovered that glycolysis was more prominent post treatment, while citrate cycle (TCA cycle) and carbon fixation pathways in prokaryotes were more enriched at the baseline (Fig. 3D).

As of the cutoff date, Patient 03 (Pt-03) exhibited the longest disease control with a PFS of 16.7 months. At the time of disease progression, fresh tumor tissue was collected and subjected to scRNA sequencing. A total of 8889 cells were processed for analysis and separated into 21 clusters via PCA analysis (Fig. 3E). Based on specific markers, nine major cell subtypes were then identified, including epithelial cells (expressing MUCL3, LINC00342 and MUC5AC), B cells (JCHAIN, IGHA1, and IGHG2), T cells (CCL5, TRBC2, and CD2), neutrophils (PDE4B, NAMPT, and CSF3R), macrophages (MPs) (HLA-DRA, HLA-DRB1, and TIMP1), plasma cells (IGHM, IGLC3, and IGLC2), fibroblasts (CXCL14, COL3A1, and POSTN), endothelial cells (ECs) (IGFBP7, MGP, and PLVAP), and mast cells (TPSB2, TPSAB1, and CAP3) (Fig. 3F and G, Supplementary Fig. 1). Potential cell–cell interaction was explored using CellPhoneDB analysis (Fig. 3H), revealing prominent interaction between fibroblast and other cell types, including ECs, MPs, and epithelial cells. Detailed cell–cell interaction analysis (Supplementary Fig. 2) suggested that CXCL12-CXCR4 pair was predicted to be involved in the connection of fibroblasts with other cell types. Upon receptor-ligand analysis, immune checkpoints TIGIT and TIM3 (TIGIT/Nectin, TIM3/Galectin 9, and LGALS9/HAVCR2) were more frequently observed compared with PD-l/PD-L1 (Supplementary Fig. 3).

## Discussion

This study provides preliminary evidence on the safety and clinical efficacy of quadruple combination with GLS-010, lenvatinib, and chemotherapy (XELOX) in AFP-elevated and HER2-negative GC patients. With favorable safety profile, this combination pattern reached ORR of 33.3% and DCR of 100%, offering new possibilities for the management of HER2 negative AFPGC.

Firstly reported by Bourreille et al*.* [16], AFPGC gained increasing attention due to its aggressive nature and poor prognosis. However, no consensus has been reached on the precise definition even after decades’ exploration [17]. Some definitions focus on histological staining, characterized by hepatic differentiation and morphological resemblance to hepatic cells and a propensity for liver metastasis (also named as hepatoid adenocarcinoma [HAC]) [18]. Others is based on serum AFP levels. In our study, a threshold of 20 ng/ml was adopted based on previous publications [19]. At this stage, treatment strategies mainly follow current guidelines for general population with no driver genes, yielding suboptimal clinical responses. Collectively, there is a pressing need to explore more effective treatment strategies for this challenging subtype.

The malignant nature and poor prognosis of AFPGC has been previously elucidated in several retrospective analyses. Li et al*.* [20] summarized clinical data from 11 published studies involving Chinese population and found that AFPGC patients presented with larger tumor size, increased lymph node and liver metastases, poorer histological types and significantly lower 3-year survival rate (11.3% vs. 60.5%), compared with non-AFPGC. Similarly, Zhan et al*.* [4] divided patients into AFP-positive and negative groups based on perioperative AFP levels (AFP > 20 ng/ml as positive) and performed a preliminary comparison on clinicopathological features, observing a higher incidence of liver metastasis and neuron invasion in positive group. Such malignant phenotype and clinical outcomes were also reported by other studies [21–23]. In our study, eight out of nine participants were firstly diagnosed with advanced disease featuring multiple distal metastases, aligning with the general understanding and previous findings. Moreover, we identified bone and peritoneal metastases in two patients each, further supporting the aggressive nature of AFPGC. In terms of efficacy analysis, a 100% DCR was achieved, with three patients (33.3%) exhibiting PR. Under current standard platinum–fluoropyrimidine chemotherapy, the mPFS for advanced GC patients without driver gene is about 6 months [24], and for AFPGC, it is expected to be even worse. Therefore, the 7.67 months of mPFS observed in our study is undoubtfully encouraging, suggesting that this combination warrants further investigation for potential clinical application.

Recent advances in next-generation sequencing (NGS) helped gain deeper insights into aggressive nature of AFPGC. Li et al*.* [25] observed a higher degree of immunosuppressive tumor microenvironment in AFPGC compared with AFP-negative cases through tissue transcriptome analysis. Besides, scRNA sequencing revealed upregulated tumor-associated pathways, including angiogenesis and epithelial-mesenchymal transition (EMT), in cells isolated from AFPGC patients compared with that from standard GC patients [26], providing the rationale of applying anti-VEGF targeted regimen in AFPGC. Recently, a whole-genome sequencing analysis on 105 AFPGC patients provided the initial landscape of somatic mutation and mutational signatures, identifying several potential therapeutic targets [27]. These findings not only outline the landscape of tumor microenvironment, but also provide theoretical basis for such combinational strategy in clinic.

In this trial, the most frequently observed AEs included fatigue (55.6%), rash (55.6%), and hand-foot syndrome (55.6%), followed by myelosuppression and gastrointestinal reactions. The majority of these AEs were classified as grade 1 or 2, with grade 3 AEs primarily observed in two patients from the 20 mg dosage group. One patient experienced grade 3 hand-foot syndrome and rash in skin of anal area, attributing to chemotherapeutic drugs and lenvatinib. Another patient suffered grade 3 decrease in neutrophil, white blood cell and platelet counts, along with grade 3 hepatic dysfunction. In addition to supportive care, a 20% reduction in oxaliplatin and capecitabine was implemented according to the protocol. With dosage adjustment and careful monitoring, these patients were able to continue their treatment. Immune-related AEs occurred in three patients, with two cases of grade 1 thyroid dysfunction and one case of pneumonia (grade 1). Hypothyroidism manifested as elevated thyroid-stimulating hormone (TSH) and decreased free triiodothyronine (FT3), free thyroxine (FT4), was successfully managed after hormone supplementation, no need for long-term medication. Grade 1 pneumonia was detected during routine radiographic screening and presented no obvious clinical symptoms. It was effectively managed with oral prednisone. Given these observations, combining lenvatinib, GLS-010 with XELOX was deemed feasible for AFPGC. With the safety and efficacy considered, 16 mg dosage was selected for subsequent expansion cohort.

Considering the distinct feature of AFPGC from typical adenocarcinoma, serum AFP level was expected to be correlated with malignant degree and prognosis. However, controversary results exist in previous investigations [28, 29]. In our study, we did not observe definite correlation between serum AFP level and tumor burden at diagnosis. With dynamic monitor of serum AFP levels during treatment, all patients experienced varying degrees of decline after first cycle treatment, with six patients exhibiting a decline more than 60%. Additionally, a rebound in AFP levels was noted at the time of disease progression, indicating that dynamic alternations in AFP levels may hold more promise in predicting prognosis than static measurements at a specific timepoint. While limited to sample size, no quantitative correlation between the extent of AFP decline and therapeutic responses was established. In the future expansion cohort, the predictive value of serum AFP alternations will be more thoroughly explored for clinical application.

Gut microbiota, as part of cancer immune microenvironment, is believed to be closely associated with tumorigenesis and treatment responses. To unveil the community alternations during treatments, stool samples were collected at baseline and after the investigational therapy (2 to 3 cycles of therapy). PCA analysis did not observe significantly separated communities, possibly attributing to the limited time interval and small sample size. However, we did observe enrichment of certain species after the treatment. *Lachnospiraceae bacterium-GAM79*, previously reported enriched in hepatobiliary cancer patients responding to anti-PD-1 therapy [30], was found significantly elevated after treatment. Additionally, a marked increase was identified in *Roseburia hominis A2-183*, a species known to decrease in ulcerative colitis (UC) and inversely correlate with disease activities [31]. With immunomodulatory effects, this species holds promise in the treatment of gut inflammatory disease [32]. Given these findings, the increased abundance after ICI-based immunotherapy was reasonable and consistent with clinical responses. Further and deeper analysis in expansion cohort is warranted to validate the role and detailed mechanism of gut microbiota during investigated treatments.

To enhance our understanding of AFPGC, scRNA sequencing was performed on tumor tissue from Pt 03 in the 12 mg dosage group, who achieved satisfied disease control with PFS of 16.7 months at the cutoff date. Through PCA analysis and unbiased cell type recognition, a total of 9 cell clusters were finally determined. Aside from epithelial cells, a certain proportion of immune cells were identified, including B cells (12.42%), T cells (10.81%), and neutrophils (9.0%). Gene expression analysis highlighted high expression of S100A8 and S100A9 in the neutrophil subgroup, aligning with the positive correlation between S100A family and invasive disease and poor prognosis [33, 34]. Moreover, inhibitory receptor KLRB1 was found strikingly expressed in T cells cluster, suggesting a possibly immunosuppressive phenotype in AFPGC. Then, cell–cell connection analysis identified that CXCL12/CXCR4 as key interaction between fibroblast and T cells as well as neutrophils. Highly involved in tumor progression and metastasis from previous research, CXCL12/CXCR4 axis is regarded as one promising therapeutic target in cancer [35, 36]. In immune checkpoint inhibitor analysis, we found that TIGIT/Nectin and TIM3/Galectin 9, rather than PD-1/PD-L1, played a prominent role in cell connections, suggesting limited efficacy of PD-1 blockade in subsequent treatment. Whether such profound expression of CXCL12/CXCR4 axis and inhibitory TIGIT and TIM3 is a general phenotype in AFPGC deserved to be determined in larger cohort, which holds great promise in developing new therapeutic targets for this subgroup.

Our study presents several limitations. Firstly, owning to the early-stage design and limited sample size, an expansion cohort with a larger sample size is imperative to further validate the clinical efficacy of this combination pattern in AFPGC. Secondly, there is no consensus on the accurate definition for AFPGC. Considering the heterogeneity among the 9 enrolled patients in this preliminary study, a more reasonable cut-off value and histological analysis are warranted for more precise medication selection. In the ongoing expansion cohort, histological and molecular information will be collected for deeper and more extensive post-data analysis.

## Conclusions

To sum up, based on our preliminary investigation, the combination of GLS-010 plus lenvatinib and chemotherapy (XELOX) demonstrated an acceptable safety profile with promising anti-tumor efficacy in HER2-negative AFPGC. Following dose escalation design, the RP2D of lenvatinib was determined. Further phase II expansion cohort is now ongoing to further validate the clinical feasibility and efficacy of this combination pattern for this specific GC subtype.

### Supplementary Information

Below is the link to the electronic supplementary material.

Supplementary file1 (PDF 929 kb)

Supplementary file2 (PDF 266 kb)

Supplementary file3 (PDF 265 kb)

## Abbreviations

AEs: Adverse events
AFP: Alpha-fetoprotein
CR: Complete response
DCR: Disease control rate
DLT: Dose limiting toxicity
DoR: Duration of response
ECOG: Eastern Cooperative Oncology Group
ECs: Endothelial cells
EMT: Epithelial-mesenchymal transition
GC: Gastric cancer
G/GEJ: Gastric and gastroesophageal junction
HAC: Hepatoid adenocarcinoma
ICD: Immunogenic cell death
ICIs: Immune checkpoint inhibitors
MPs: Macrophages
MTD: Maximum tolerated dose
NCI-CTCAE: National Cancer Institute Common Terminology Criteria for Adverse Events
NGS: Next generation sequencing
ORR: Overall response rate
OS: Overall survival
PD: Progressive disease
PD-1: Programmed cell death protein 1
PD-L1: Programmed cell death protein ligand 1
PFS: Progression-free survival
PR: Partial responses
RECIST: Response evaluation criteria in solid tumors
RP2D: Recommended phase II dose
SAE: Serious adverse events
SD: Stable disease
TME: Tumor microenvironment
TRAEs: Treatment-related adverse events
ULN: Upper limit of normal

## Declarations

### Availability of data and materials

The datasets used and/or analyzed during the current study are available from the corresponding author on reasonable request.

### Trial registration

ClinicalTrials.gov Identifier: NCT05221775.
