# Supplementary figures and images for: Combinational zimberelimab plus lenvatinib and chemotherapy for alpha-fetoprotein elevated, advanced gastric cancer patients (AFPGC): a phase 1 dose-escalation study
Source: Cancer Immunol Immunother. 2024 Jun 4;73(8):154. doi: 10.1007/s00262-024-03743-0 (PMC11150360; doi:10.1007/s00262-024-03743-0)

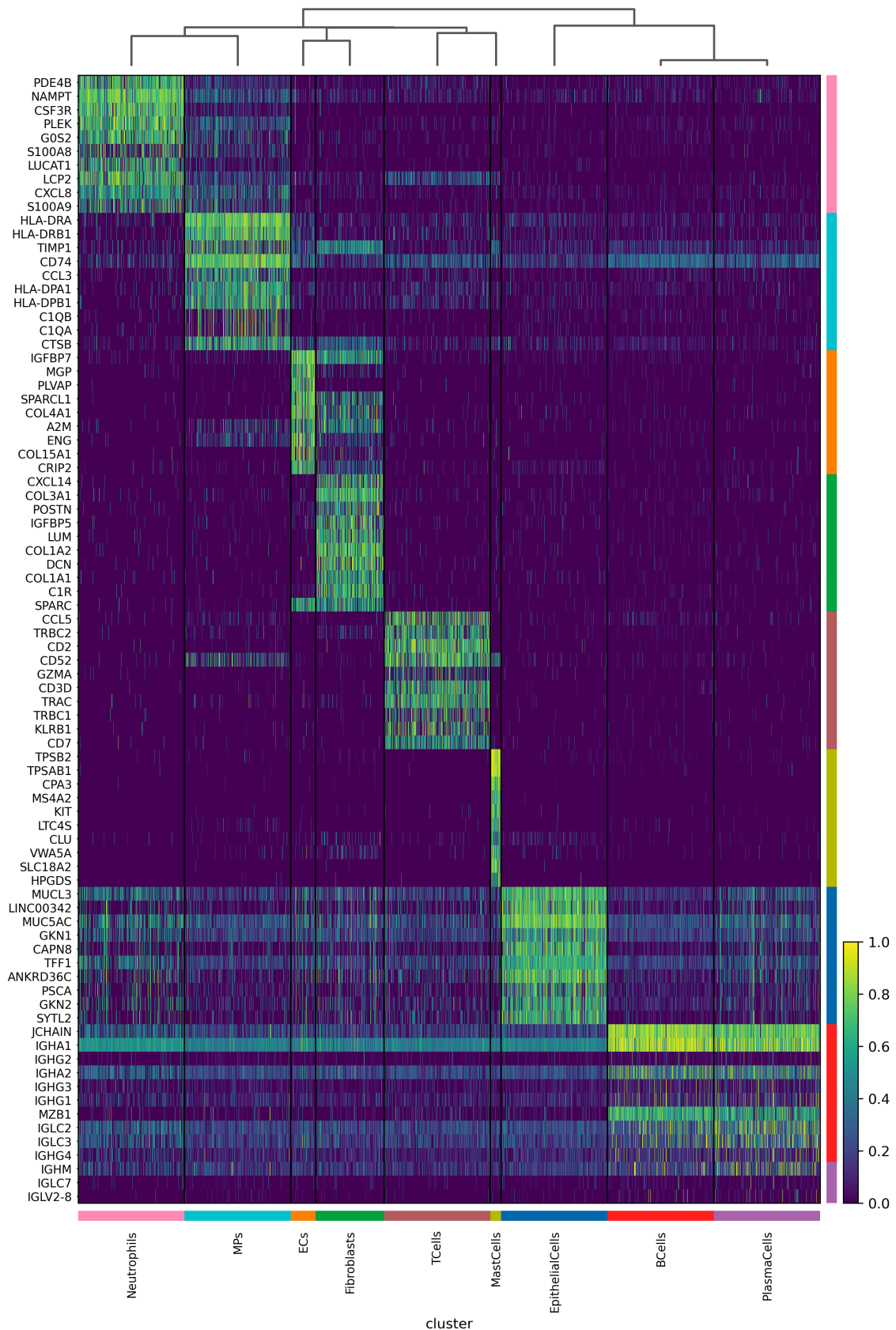

**Supplementary Figure 1 Heatmap for differentially expressed genes in cell clusters.**

Supplement: Supplementary file 1 — Supplementary file1 (PDF 929 kb) [file 262_2024_3743_MOESM1_ESM.pdf]
