# Supplementary material for: Combinational zimberelimab plus lenvatinib and chemotherapy for alpha-fetoprotein elevated, advanced gastric cancer patients (AFPGC): a phase 1 dose-escalation study
Source: Cancer Immunol Immunother. 2024 Jun 4;73(8):154. doi: 10.1007/s00262-024-03743-0 (PMC11150360; doi:10.1007/s00262-024-03743-0)
Supplement: Supplementary file 3 — Supplementary file3 (PDF 265 kb) [file 262_2024_3743_MOESM3_ESM.pdf]

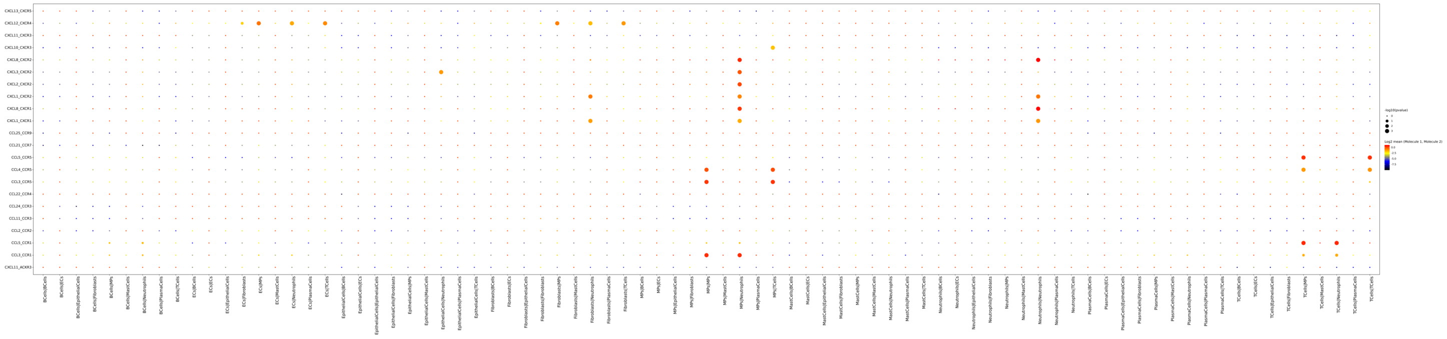

Supplementary Figure 3. Dot plot of predicted ligand-receptor interactions (checkpoint inhibitors) between different subsets of cell clusters in tumor sample.
